# Accuracy of the Fluorescence-Activated Cell Sorting Assay for the Aquaporin-4 Antibody (AQP4-Ab): Comparison with the Commercial AQP4-Ab Assay Kit

**DOI:** 10.1371/journal.pone.0162900

**Published:** 2016-09-22

**Authors:** Jiwon Yang, Sung Min Kim, Yoo-Jin Kim, So Young Cheon, Boram Kim, Kyeong Cheon Jung, Kyung Seok Park

**Affiliations:** 1 Department of Neurology, Gachon University, Gil Medical Center, Incheon, Korea; 2 Department of Neurology, Seoul National University, College of Medicine, Seoul, Korea; 3 Department of Pathology, Seoul National University, College of Medicine, Seoul, Korea; Medizinische Universitat Innsbruck, AUSTRIA

## Abstract

**Background:**

The aquaporin-4 antibody (AQP4-Ab) is a disease-specific autoantibody to neuromyelitis optica (NMO). We aimed to evaluate the accuracy of the FACS assay in detecting the AQP4-Ab compared with the commercial cell-based assay (C-CBA) kit.

**Methods:**

Human embryonic kidney-293 cells were transfected with human aquaporin-4 (M23) cDNA. The optimal cut off values of FACS assay was tested using 1123 serum samples from patients with clinically definite NMO, those at high risk for NMO, patients with multiple sclerosis, patients with other idiopathic inflammatory demyelinating diseases, and negative controls. The accuracy of FACS assay and C-CBA were compared in consecutive 225 samples that were collected between January 2014 and June 2014.

**Results:**

With a cut-off value of MFIi of 3.5 and MFIr of 2.0, the receiver operating characteristic curve for the FACS assay showed an area under the curve of 0.876. Among 225 consecutive sera, the FACS assay and C-CBA had a sensitivity of 77.3% and 69.7%, respectively, in differentiating the sera of definite NMO patients from sera of controls without IDD or of MS. Both assay had a good specificity of 100% in it. The overall positivity of the C-CBA among FACS-positive sera was 81.5%; moreover, its positivity was low as 50% among FACS-positive sera with relatively low MFIis.

**Conclusions:**

Both the FACS assay and C-CBA are sensitive and highly specific assays in detecting AQP4-Ab. However, in some sera with relatively low antibody titer, FACS-assay can be a more sensitive assay option. In real practice, complementary use of FACS assay and C-CBA will benefit the diagnosis of NMO patients, because the former can be more sensitive among low titer sera and the latter are easier to use therefore can be widely used.

## Introduction

Neuromyelitis optica (NMO) is an inflammatory demyelinating disease of the central nervous system [1]. Although NMO has long been considered to be a variant of multiple sclerosis (MS), the discovery of the NMO-specific autoantibody against aquaporin-4 (AQP4-Ab) identified NMO as a separate disease from MS [1]. NMO is also distinct from MS in that the former is more predominant in females (up to 9:1) [2], is associated with more severe attacks in the optic nerve and the spinal cord, and can be treated with immune suppressive agents or B cell depletion [3]. The prevalence of NMO was reported to be up to 4.4 per 10^5^ individuals [4].

Though NMO usually manifests as severe optic nerve and spinal cord involvement [5, 6], a considerable number of patients with NMO show only isolated symptoms related to either the optic nerve, spinal cord, or even the brain (limited forms) in the early stage of the disease [7]. Therefore, besides the clinical diagnosis by physicians, testing for the AQP4-Ab in the serum of patients can often be essential for diagnosing NMO.

Diverse assays for accurately determining the AQP4-Ab status of a patient have been developed [8, 9]. Among these diverse assay methods, cell-based serum assays (CBAs) that use live cells expressing human AQP4 (microscopy or fluorescence-activated cell sorting-based methods) are recommended by the International Panel for NMO as the optimized methods for autoantibody detection [10]. However, the CBAs that use live cells are currently only performed in a few laboratories, mostly because of its complicated methods [11]. To overcome the limited availability of CBAs, a commercial assay kit that uses pre-fixed cells on chips, rather than live cells, has recently been introduced [9].

In the present study, we aimed to report the accuracy of the CBA that utilizes fluorescence-activated cell sorting (FACS) compared to a commercial CBA (C-CBA) kit in detecting AQP4-Ab.

## Methods

### Patients

**Fig 1:**
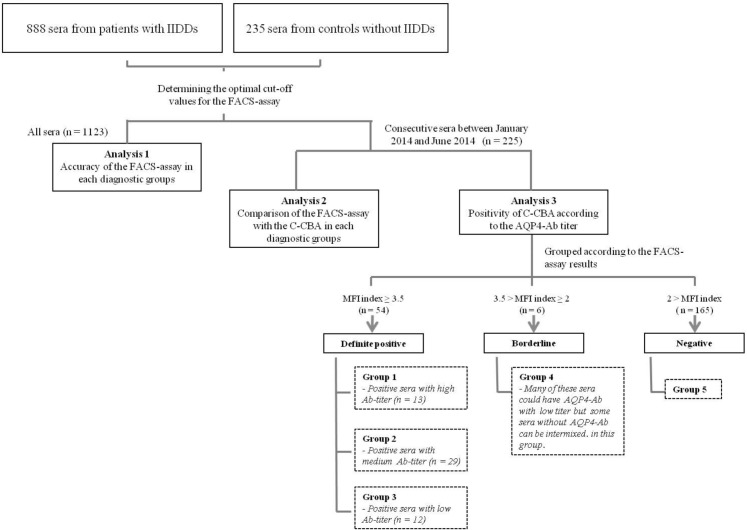
Flow chart illustrating the process of subject selection and analysis. For the analysis of the accuracy of the FACS assay, all 1123 serum samples were used. To compare the accuracy of the FACS assay with the C-CBA kit, 225 serum samples (54 FACS-positive, 6 FACS-borderline, and 165 FACS-negative) were used. These 225 samples were further classified into groups 1–5 according to the MFIi of the FACS assay: Group 1 = AQP-4 Ab positive sera with high titer of MFIi (≥ 50), Group 2 = AQP-4 Ab positive sera with medium titer of MFIi (10 ≤ MFIi < 50), Group 3 = AQP-4 Ab positive sera with low titer of MFIi (3.5 ≤ MFIi < 10), Group 4 = sera with borderline MFIi (2 ≤ MFIi < 3.5), Group 5 = AQP-4 Ab negative sera (MFI < 2). MFIi values were considered to be significant only when the MFI ratio is ≥2). The researchers who performed the C-CBA were blind to the results of the FACS assay. Abbreviations: AQP4-Ab = aquaporin-4 antibody, C-CBA = commercial cell-based assay, FACS = fluorescence-activated cell sorting, FACS-positive = tested positive in the FACS assay, FACS-negative = tested negative in the FACS assay, FACS-borderline = tested borderline in the FACS assay, IIDD = idiopathic inflammatory demyelinating disease, MFIi = mean fluorescence intensity index.

In total, 1123 consecutive serum samples collected from patients (493 men, mean age: 47.0 years, range: 8.5 to 97.9 years) who were suspected as having idiopathic inflammatory demyelinating diseases (IIDDs) of the central nervous system such as MS [12], definite NMO with the exception of AQP4 antibody status [6], NMO spectrum disorder (NMOSD) [13], acute disseminated encephalomyelitis (ADEM) [14], acute transverse myelitis [15], optic neuritis (ON) [16], or clinically isolated syndrome of the brain [17] were screened; all had visited the Seoul National University Hospital or Seoul National University Bundang Hospital between September 1, 2009, and February 28, 2014. Two hundred thirty-five patients who had neurological diseases other than IIDDs, including vascular, tumorous, degenerative, infectious, metabolic, or hereditary diseases, or peripheral neuropathy, were also included as a control group to determine the cut-off value of the quantitative FACS assay for AQP4-Ab (Fig 1).

### FACS assay

The assay for the AQP4-Ab was performed by S.M.K. and Y.J.K. at Seoul National University Hospital using FACS, with minor modifications from methods published previously [9]. Human embryonic kidney cells containing the SV40 large T antigen (HEK-293T) were transiently transfected with plasmid containing Ds-Red-tagged human AQP4 cDNA for the M23 isoform (Clontech, from Dr. Waters at John Radcliffe Hospital). The cells (5 × 10^6^) were incubated in 2 mL of growth medium for 1 day before transfection. Then, 4 μg of AQP4-cDNA were diluted in 250 μL of serum-free media, mixed gently with 10 μL of Lipofectamine (Life Technologies, USA), and incubated at 37°C for 24 h. The cells were washed with FACS buffer, and 2 μL of the collected serum samples (from patients or controls) were added to a FACS tube containing 5 × 10^5^ cells. The mixture containing the AQP4-expressing cells and serum was incubated for 1 h at 4°C, washed with FACS buffer, and centrifuged at 1800 rpm for 3 min. Fluorescein isothiocyanate (FITC)-conjugated secondary antibodies against human immunoglobulin G were added to this mixture and incubated for 30 min at 4°C. Plasma protein (diluted 1:30) obtained via the therapeutic plasmapheresis of a patient seropositive for NMO (52 years old female with recurrent optic neuritis and myelitis, tested positive at the John Radcliffe Hospital, Oxford, UK, by using a cell-based assay, as has been previously described [18]) and a healthy control subject were used as positive and negative control, respectively. The mean fluorescence intensity (MFI) values for FITC were measured as the binding of human immunoglobulin G to the AQP4 that was expressed at the cell surface. The MFI index (MFIi) was determined according to the following formula: MFIi = (MFI of patients–MFI of healthy control)/(MFI of the positive control–MFI of the healthy control) [19]. However, MFIi can falsely overestimate FACS-positive results when the difference between MFI of the positive control and MFI of healthy control become small. Therefore, we used MFI ratio (MFIr) to overcome the possible pitfall of MFIi. The MFIr was calculated according to the following formula: MFIr = MFI of the patients/MFI of the healthy control. Then, MFIi values were considered to be significant only when the MFIr cut-off value of ≥2. The receiver operating characteristic (ROC) curve analysis was performed using 406 serum samples (171 serum samples from patients with definite NMO and 235 samples from patients without IIDDs). The accuracy of the FACS assay was evaluated using the assay positive rate among the diverse diagnostic groups.

### Diagnostic classification of the patients

Patients were classified based on the diagnostic criteria for definite NMO with the exception of AQP4 antibody status [6], high risk for NMOSD other than definite NMO [13], MS [12], ADEM [14], or other IIDDs including acute transverse myelitis [15], ON [16], or clinically isolated syndrome of the brain [17]. Two neurologists (S.M.K. and J.W.Y.) independently assessed the diagnosis of the patients according to the published criteria [6, 12–14, 20, 21]. Discussion and reassessment of the clinical records were performed for any diagnostic disagreement until consensus.

### Comparison of the FACS assay with the C-CBA kit

Consecutive 225 serum samples in a certain period of time (between January 2014 and June 2014) were tested for the AQP4-Ab using the C-CBA kit, having fixed AQP4-transfected HEK cells on slides as an antigenic substrate (Euroimmune kit order number: FA1128-1010-50, Euroimmune kit order number: FA1128-1005-50, Lot number: F160225CB, Lubeck, Germany). Two observers (S.Y.C. and B.K.), who were blind to the clinical information and FACS assay results of the serum samples, independently classified the samples as positive or negative according to the manufacture’s protocol [22]. Inconsistent C-CBA test results between these two observers were classified as borderline results (Fig 1).

### Standard protocol approval, registration, and patient consent

This study was approved by the Institutional Review Board of Seoul National University Hospital and Seoul National University Bundang Hospital (IRB number: H 1012-023-317 and B-1007-105-401). All patients provided written informed consent.

## Results

### Accuracy of the FACS assay

**Fig 2:**
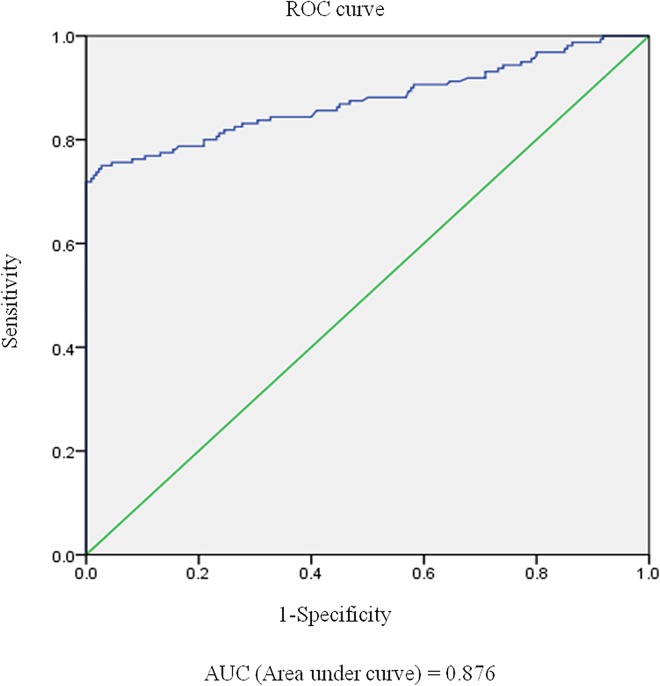
Results of the ROC analysis. The ROC analysis showed that the MFIis for the FACS revealed an area under the curve of 0.876 when detecting sera from patients with definite NMO. Abbreviations: FACS = fluorescence-activated cell sorting, MFIi = mean fluorescence intensity index, NMO = neuromyelitis optica, ROC = receiver operating characteristic.

**Fig 3:**
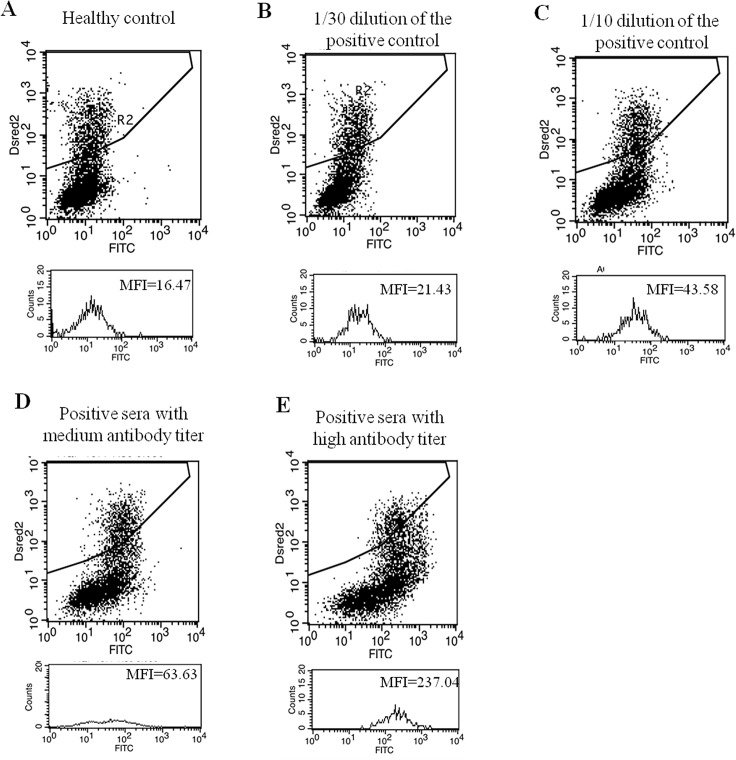
Examples of the FACS data. The FACS data for healthy control, positive control with diverse dilution concentrations, and positive test results are shown.

The ROC analysis showed that the MFIi obtained using the FACS assay had an area under the curve of 0.876 when detecting sera from patients with definite NMO (Fig 2). Fig 3 illustrates our FACS data for the healthy control, positive control with diverse concentrations, and positive test results. The MFIi values for each diagnostic group are summarized in Table 1. As the maximal MFIi values in the control group without IIDDs (n = 235) and the MS group (n = 88) were 1.99 and 3.20, respectively, we tested the accuracy of the FACS assay using the two different cut-off values of ≥2 and ≥3.5, respectively (Table 1). With an MFIi cut-off value of ≥2, the FACS assay showed positive test results in 70.2% of the serum samples from patients with definite NMO, without showing any positive test results among the serum samples from patients without IIDDs. However, with an MFIi cut-off value of ≥2, one serum sample from the MS group (1.1%) could be classified as positive, which is false-positive test result. With an MFIi cut-off value of ≥3.5, 66.1% of the serum samples from patients with definite NMO showed positive test results, while none of the serum samples from either the MS group or controls without IIDDs tested positive. Therefore, we classified sera with MFIis of ≥3.5 as definitely AQP4-Ab-positive and sera with MFIis in between 2 and 3.5 (2 ≤ MFIi < 3.5) as having a high possibility of being AQP4-Ab-positive (borderline). As a result, the FACS assay with the MFIi of ≥3.5 showed the sensitivity and specificity as 77.3% (CI 0.53–0.82) and 100% (CI 0.93–1.00). Interestingly, among the 171 serum samples from the patients with definite NMO, 16 samples were obtained within 1 month of disease onset and before the use of intravenous methylprednisolone or plasmapheresis, and most of these samples (15/16) showed MFIis ≥3.5 (data not shown).

**Table 1:** Number of AQP4-Ab-positive serum samples in each diagnostic group according to the mean fluorescence intensity index cut-off values.

	Definite NMO (n = 117)	High risk for NMO (n = 384)	MS (n = 88)	ADEM (n = 30)	Other IIDDs (n = 215)	Controls without IIDDs (n = 235)	Total (n = 1123)
*Positive (MFIi ≥ 3.5)	113 (66.1%)	104 (27.1%)	0 (0%)	0 (0%)	3 (1.4%)	0 (0%)	219 (19.5%)
*Borderline (3.5 > MFIi ≥ 2.0)	7 (4.1%)	11 (2.9%)	1 (1.1%)	0 (0%)	3 (1.4%)	0 (0%)	22 (2.0%)

Abbreviations: AQP4-Ab = aquaporin-4 antibody, IIDD = idiopathic inflammatory demyelinating disease, NMO = neuromyelitis optica, MS = multiple sclerosis, ADEM = acute disseminated encephalomyelitis, MFIi = mean fluorescence intensity index.

*MFIi values were considered to be significant only when the MFI ratio is ≥2)

### Comparison of the FACS assay with the C-CBA kit

Consecutive 225 serum samples in a certain period (6 months) were also tested using the C-CBA to compare the accuracies of the two assays. The positivity of FACS-assay and C-CBA in each diagnostic group was summarized in Table 2, and the overall positivity of these assays was 24% and 19.5%, respectively. As some of the NMOSD patients with AQP4-Ab may not meet the definite diagnostic criteria for NMO [23], nor does not have clinical features of NMO (high risk group) [24] in their early disease stages, we have measured the sensitivity and specificities of assays in differentiating sera of definite NMO (highly AQP4-Ab positive) from those of controls without IDD or MS (both of these groups should not have AQP4-Ab).

**Table 2:** Comparison of the FACS assay with the commercial cell-based assay kit in diverse disease group.

	Definite NMO (n = 46)	High risk for NMO (n = 47)	MS (n = 10)	ADEM (n = 10)	Other IIDDs (n = 53)	Controls without IIDDs (n = 59)	Total (n = 225)
FACS-assay positive	34 (73.9%)	20 (42.6%)	0 (0%)	0 (0%)	0 (0%)	0 (0%)	54 (24%)
C-CBA positive	30 (65.2%)	13 (27.7%)	0 (0%)	0 (0%)	1 (1.9%)	0 (0%)	44 (19.5%)

Abbreviations: FACS = fluorescence-activated cell sorting, AQP4-Ab = aquaporin-4 antibody, C-CBA = commercial cell-based assay.

The C-CBA showed sensitivity of 69.7% (CI 0.53–0.82) and specificity of 100% (CI 0.93–1.00) for sera from patients with definite NMO, which was comparable with the FACS-assay. The results were grouped according to the MFIi titer of the FACS assay and the C-CBA showed relatively high positive rate among sera with high MFIis (group 1, 100%) and medium MFIis (group 2, 79.3%). However, among the FACS-positive sera with low MFIis (group 3), the test positivity of the C-CBA was low (50.0%). In total, the positivity of the C-CBA among FACS-positive sera was 77.8%. Among the borderline sera (group 4), the positivity of the C-CBA was 33.3%. None of the FACS-negative sera tested positive in the C-CBA (Table 3). Fifty-six samples tested positive either in the FACS assay or the C-CBA and 14 discrepant results were observed between the C-CBA and the FACS assay (12 positive only in the FACS assay and 2 positive only in the C-CBA) (S1 Table).

**Table 3:** Results of the commercial cell-based assay kit according to the FACS assay grouped by mean fluorescence intensity index titer.

Groups according to the FACS assay	C-CBA			Total
	Positive	Borderline	Negative	
1 (high MFIi titer positive, n = 13)	13 (100%)	0	0	13 (100%)
2 (medium MFIi titer positive, n = 29)	23 (79.3%)	2 (6.9%)	4 (13.8%)	29 (100%)
3 (low MFIi titer positive, n = 12)	6 (50%)	0	6 (50%)	12 (100%)
4 (borderline, n = 6)	2 (33.3%)	1 (16.7%)	3 (50%)	6 (100%)
5 (negative, n = 165)	0	6 (3.6%)	159 (96.4%)	165 (100%)
Total	44	9	172	225

Abbreviations: FACS = fluorescence-activated cell sorting, C-CBA = commercial cell-based assay, MFIi = mean fluorescence intensity index.

## Discussion

In the present study, we demonstrated the diagnostic sensitivity and specificity of our in-house FACS assay and showed its advantages over the C-CBA kit in detecting serum AQP4-Ab with low titers.

We showed that our in-house FACS assay, which uses unfixed live cells, has a better sensitivity and specificity than the C-CBA kit. Our results are in accordance with a previous study by Waters et al. [9], which reported the sensitivities of the FACS assay and C-CBA as 76.7% and 68.3%, respectively, in detecting patients with NMOSD. Our findings are also consistent with a study by Jiao et al. [25], which reported the positivity of the FACS assay and C-CBA as 56% and 48%, respectively. In addition to the results of previous studies, we found that the relatively low positivity of the C-CBA was most profound in sera with relatively low AQP4-Ab titers. This finding is important because it implies that patients with NMOSD with relatively low AQP4-Ab titers, perhaps due to low disease activity, tight immune suppression, or plasmapheresis, could easily be misdiagnosed as AQP4-Ab-negative (false negative) patients with the C-CBA kit. In our study, among the 54 serum samples that tested positive in the FACS assay, 11(20.4%) of them tested negative (false negative) when using the C-CBA. In addition, 14 samples showed discrepant results between the C-CBA and the FACS assay. Patients that tested positive only in the FACS assay were all definite NMO or high risk for NMO and their samples were taken when the patients were under immunosuppressant, high dose steroid or plasmapheresis. Regarding 2 samples that tested positive only in the C-CBA, one patient was diagnosed with definite NMO and the rest with other IIDDs and they tested borderline in the FACS-assay. We speculated that this could be due to the cytotoxicity of the AQP4-Ab to the live cells. This phenomenon has been shown also in CBA using live cells in a previous review paper [26]. Therefore, in interpreting the results of the FACS-assay, it seems necessary to check the dot plot as well as the MFI values.

The relatively lower sensitivity of the C-CBA compared to FACS, especially in samples with low AQP4-Ab titers, could be attributable to several factors. First, the currently available C-CBA adapts pre-fixed HEK-293 cells [22], and, as emphasized by Fujihara et al. [27], this pre-fixation in the C-CBA will decrease the sensitivity of the assay results by increasing non-specific binding to the cells. Second, a previous study showed that the AQP4-Ab had a greater binding affinity to the M23 than to the M1 AQP4 isoform, which was caused by higher orthogonal array of particle formation of M23 than M1. However, the cells fixed in the C-CBA kit expressed only the AQP4 M1 isoform [22, 28], and thereby could have limited orthogonal array of particle formation [29]. Lastly, the results of the C-CBA are interpreted by visually observing the immunofluorescence signals with a microscope, which could be highly observer-dependent and lack objective quantification of the results. On the contrary, our in-house FACS assay used live unfixed cells that expressed AQP4-M23 isoforms, and the results of the FACS assay are quantified as an MFI score. For the abovementioned reasons, we believe that the FACS assay may produce more sensitive assay results compared to the C-CBA, especially in serum samples with low antibody titers.

Our FACS assay showed negative test results in 29.8% of the patients with definite NMO. This may be because a considerable number of our patients underwent serum sampling after plasmapheresis or rituximab therapy or while they were in a chronic remission stage, all of which will lower the serum titer of the autoantibodies. Interestingly, among the 171 serum samples from patients with definite NMO, 16 samples were obtained in the acute stage (within 1 month of onset) and before the use of immune suppressants or plasmapheresis. The positivity of our FACS assay among acute-stage, treatment-naïve sera was as high as 93.8% (15/16). Therefore, the diagnostic accuracy of the FACS assay can be greatly improved by obtaining samples while patients are in the acute stage, before any immunosuppressive treatment or plasmapheresis.

We also showed that adopting the category of “borderline results” can have advantages over the dichotomous classification of positive or negative. In this study, 7 (4.1%) of the samples from patients with definite NMO and 11 (2.9%) of the samples from patients who were at high risk for NMO were considered to have “borderline results.” Some samples from patients with NMO can have low antibody titers after immuno-suppressive treatments such as B cell depletion or plasmapheresis. Therefore, the samples that were categorized as borderline should be re-tested for a confirmative diagnosis and to improve the diagnostic sensitivity.

Although we showed that the FACS assay has a higher sensitivity than the C-CBA in detecting the AQP4-Ab, the C-CBA also has its advantages because it is a simple, convenient, and ready to use method. The C-CBA also has good sensitivity in detecting sera with medium-high AQP4-Ab titers and high specificity. Moreover, the special education and experience that are necessary to skillfully operate and maintain the FACS device are not needed for the C-CBA. Therefore, we believe that complementary use of the C-CBA and FACS assay will optimize the diagnostic yield of AQP4-Ab assays in clinical practice.

Two of our patients “Other IIDD” group were tested positive for the FACS-assay. We believe that all of 2 patients could be in the early stage of NMO. Because one patient had recurrent myelitis without brain lesions and the other patient had brainstem lesions without having typical PVWM lesions of MS, all of which can be observed in patients with NMO. Moreover, we have previously shown that majority of patients with NMO can manifest as a limited manifestations in their early stage of disease.

Our study and our in-house FACS assay have some limitations. First, we could not test all of the 1123 samples simultaneously with the FACS and C-CBA methods, mostly due to funding limitations. However, because we compared the accuracies of the FACS and C-CBA in the individual groups with different MFIis (i.e., groups 1 to 5), we do not believe that inability to test all samples with both assays interfered with our results showing that the positivity of C-CBA can be relatively low among sera with low antibody titers. Second, although we tested all the consecutive samples in a certain period of 6 months to avoid selection bias, we cannot completely rule out the possibility that there might be a small selection bias because we did not test all the samples. Third, to exclude minor positivity of AQP-4 Ab in patients with other IIDDs and discrepancy between the C-CBA and the FACS assay, a third assay would be helpful to determine definite and accurate results. Fourth, sera with autoantibodies associated with connective tissue disease are known to be particularly ‘sticky’ in the FACS assay, and can cause false-positive results by non-AQP-4 Ab-specific binding. However, we did not check for those antibodies in sera with AQP-4 Ab positive. Finally, our FACS assay used live cells that expressed human AQP4. These live cells require special skills, experience, and time in order to properly maintain and handle them, which are the major obstacles stopping more labs from using the FACS assay to detect the AQP4-Ab.

In conclusion, our in-house FACS assay is a useful method with a high sensitivity in detecting the AQP4-Ab. In addition, it has its advantages over the C-CBA kit, mostly in detecting samples with low AQP4-Ab titers and samples with a high probability of having the AQP4-Ab (borderline results). Understanding the advantages and pitfalls of these diverse types of AQP4-Ab assay methods is crucial in clinical practice and for researching NMOSD.

## Supporting Information

S1 Table Raw data of 166 sera with idiopathic inflammatory demyelinating disease tested in both FACS-assay and the commercial cell-based assay kit. Fifty-six samples tested positive either in the FACS-assay or the C-CBA. Among them 14 serum samples showed discrepant results between two assays: 12 were positive only in the FACS-assay and 2 were positive only in the C-CBA. Abbreviations: FACS = fluorescence-activated cell sorting, C-CBA = commercial cell-based assay, NMO = Neuromyelitis optica, IIDD = idiopathic inflammatory demyelinating disease.
(XLSX)
