# Correction: Accuracy of the Fluorescence-Activated Cell Sorting Assay for the Aquaporin-4 Antibody (AQP4-Ab): Comparison with the Commercial AQP4-Ab Assay Kit

**DOI:** 10.1371/journal.pone.0180379

**Published:** 2017-06-22

**Authors:** Jiwon Yang, Sung Min Kim, Yoo-Jin Kim, So Young Cheon, Boram Kim, Kyeong Cheon Jung, Kyung Seok Park

The affiliation for the last author is incorrect. Kyung Seok Park is not affiliated with #1 but with #2 Department of Neurology, Seoul National University, College of Medicine, Seoul, Korea and #4 Department of Neurology, Seoul National University Bundang Hospital, Seongnam, Korea.
